# Interleukin 10 drives *Staphylococcus aureus* imprinting and vaccine failure in murine models via antibody glycosylation

**DOI:** 10.1172/JCI187055

**Published:** 2024-12-16

**Authors:** Victor J. Torres

**Affiliations:** Department of Host-Microbe Interactions, St. Jude Children’s Research Hospital, Memphis, Tennessee, USA.

## Abstract

Despite many attempts, there is currently no approved vaccine to prevent *Staphylococcus aureus* infections. Preclinical vaccination models have failed to predict vaccine efficacy in humans as *S*. *aureus* exposure in humans imprints an immune response that is lacking in naive animals. In this issue of the *JCI*, Tsai and colleagues identify the cytokine IL-10 as the driver of humoral imprinting by *S*. *aureus*. Upon vaccination, *S*. *aureus*–experienced animals produced copious levels of IL-10, resulting in the hyper-α2,3 sialylation of antibodies, which interfered with the phagocytic-promoting properties of the vaccine-elicited anti–*S*. *aureus* antibodies. These findings correlate with the observation that hyperproduction of IL-10 in humans also induces hyper-α2,3 sialylation of antibodies and provide a possible mechanism for previous vaccine failures.

## Pathogenesis versus commensalism of *Staphylococcus aureus*

*Staphylococcus aureus* (*S. aureus*) is a formidable Gram-positive bacterium that is the second leading human pathogen responsible for the most global deaths attributable to and associated with bacterial antimicrobial resistance (1). *S*. *aureus* is responsible for a large number of invasive life-threatening infections, including bacteremia, necrotizing pneumonia, endocarditis, and osteomyelitis, and it is considered a serious threat by both the CDC (2) and WHO (3). The pervasiveness of this organism is partly due to the emergence of multidrug-resistant clones, like methicillin-resistant *S*. *aureus* (MRSA) (4), which severely restricts antimicrobial treatment options.

Due to the plethora of host tissues that *S*. *aureus* infects and colonizes, the pathogenesis of this bacterium is complex. To thrive in the mammalian host, *S*. *aureus* deploys an armamentarium of virulence factors composed of potent lytic pore-forming toxins, enzymes with diverse substrates, a repertoire of surface adhesins involved in attachment and biofilm formation, and a large array of secreted immunomodulators that inhibit processes involved in both innate and adaptive immunity (5–7).

While much of the existing *S*. *aureus* research has focused on pathogenesis to develop preventive strategies, the most common interaction of *S*. *aureus* with the human host occurs during commensalism where the bacterium colonizes the nares, skin, and the gastrointestinal tract (GI) (8–10). It is estimated that approximately 30% of the human population carries this bacterium as a commensal (9). Moreover, exposure to *S*. *aureus* takes place early in life (11) and humans harbor anti–*S*. *aureus* antibodies against most virulence factors (12). However, this response is insufficient to protect from subsequent *S*. *aureus* infections, as recurrent infections are quite common.

## Vaccine failures

*S. aureus* belongs to a select club of fastidious pathogens that have eluded vaccine development. While scientists have been able to elicit potent immune responses in preclinical models of infection against myriads of *S*. *aureus* antigens, translation of these results to humans has been unsuccessful. It is estimated that over thirty human clinical trials have failed to identify protective passive- and/or active-immunization regimens (13). This Herculean effort, and investment, highlights the unmet need for anti–*S*. *aureus* vaccines and/or biologics as well as the challenges we face developing them.

The large assortment of virulence factors produced by *S*. *aureus* has made it difficult to identify the select few that could be developed into manufacturable vaccine antigens. Compounding this, there are at least two additional major limitations worth noting. First, *S*. *aureus* clinical isolates exhibit exquisite tropism toward humans with many powerful virulence factors ineffective in preclinical animal models. Second, in contrast to humans that are exposed to *S*. *aureus* shortly after birth, most preclinical models are naive to *S*. *aureus*. The impact of prior exposure on vaccine efficacy is an important topic and one that has been mostly ignored in the field.

## IL-10 drives vaccine failure in preinfected mice

Human exposure to *S*. *aureus* triggers the development of immunity, imprinting an adaptive immune response that is lacking in most current preclinical models. In a recent study, Tsai et al. explored the impact of prior infection on the efficacy of vaccination with the iron-regulated surface determinant B (IsdB) in mice (14). IsdB is a conserved surface protein in *S*. *aureus* that is involved in iron acquisition (15) and pathogenesis (16). As with many antigens, IsdB elicits robust and protective responses in naive mice (14, 17). However, in a randomized placebo-controlled human trial in patients undergoing cardiothoracic surgery, IsdB vaccination failed to prevent *S*. *aureus* bacteremia and/or deep wound infection (18). Tsai et al. demonstrated that while vaccination of naive animals elicited the expected protective response, vaccination of previously infected mice failed to protect due to the elaboration of antibodies that are impaired in opsonophagocytosis and opsonophagocytic killing (OPK), processes essential for pathogen clearance (14). Interestingly, the reduced OPK activity was linked with the α2,3 sialylation of the anti-IsdB antibodies, a modification that blocks engagement of the Fcγ receptors on phagocytes (19).

**Figure 1:**
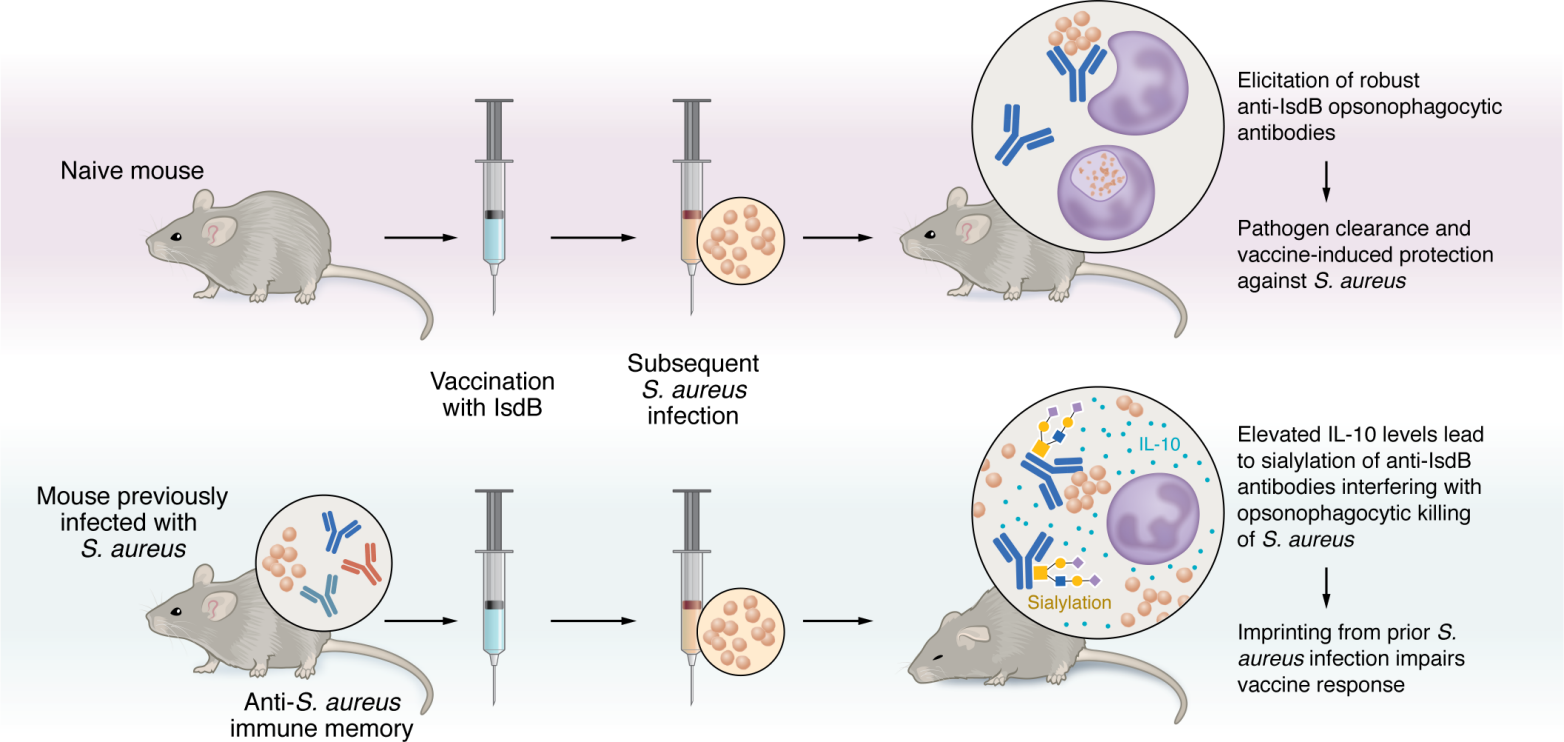
Imprinting due to prior *S*. *aureus* infection blocks vaccine efficacy. In the naive host, vaccination with IsdB elicits opsonic antibodies that promote phagocyte-mediated eradication of *S*. *aureus*. In contrast, IsdB vaccination of mice previously infected with *S*. *aureus* triggers a recall response accompanied with copious levels of IL-10, which results in the sialylation of the anti-IsdB antibodies, rendering these antibodies ineffective at promoting OPK.

In this issue of the *JCI*, Tsai et al. reports elevated levels of IL-10 in animals previously infected with *S*. *aureus* that were subsequently vaccinated with IsdB compared with those without prior infection (20). IL-10 is a multifactorial cytokine that has been linked to increased mortality in patients with *S*. *aureus* bacteremia (21). Animals infected with *S*. *aureus* had elevated levels of B10 lymphocytes, a type of B cell responsible for the production of IL-10. Mechanistically, IL-10 induced the expression and production of St3gal4 and St6gal2, sialyltransferases that mediated the α2,3 sialylation on the anti-IsdB antibodies. Importantly, protection could be restored in mice that were transferred anti-IsdB antibodies treated with an α2,3 neuraminidase, which removed antibody sialylation and restored OPK. Thus, restoration of antibody-mediated OPK activity was sufficient to overcome vaccine failure in mice. Of note, IL-10 also induced α2,3 sialylation of antibodies elicited upon IsdA and MntC vaccination. Thus, prior *S*. *aureus* infection induces IL-10, which limits the vaccine efficacy of at least three different surface antigens (Figure 1).

To provide a link between the mouse studies and the human scenario, Tsai et al. (20) examined the glycosylation state of antibodies from healthy individuals and patients with cystic fibrosis (CF), who are known to harbor higher amounts of IL-10 (22). In support of the murine data, antibodies from patients with CF were hyper-α2,3 sialylated compared with antibodies from healthy controls. Moreover, removal of α2,3 sialylation from naturally occurring human anti-IsdB antibodies enhanced OPK of *S*. *aureus*. From these findings, we can wonder if the observed linkage between IL-10 and mortality in patients with *S*. *aureus* bacteremia (21) could be due to hyper-α2,3 sialylation of natural anti–*S*. *aureus* antibodies rendering them ineffective.

## Limitations and outlook

Tsai et al. provides a key missing link between vaccine efficacy in naive and pathogen-experienced animals (20). Several questions arise regarding the implications of these findings for future vaccine strategies. First, the Tsai et al. studies focused on the surface antigen IsdB and several other surface antigens (20). But what would be the effect of α2,3 sialylation of antibodies against secreted antigens? It is tempting to speculate that glycosylation would play a minor role in antibodies that directly bind and block the function of secreted virulence factors compared with antibody-antigen interactions that depend on Fcγ engagement to promote OPK. This proposition likely explains the observed efficacy in a recent vaccination study using a toxoid in minipigs (23). Swine are a reservoir of *S*. *aureus* and contain natural anti-bacterial antibodies (23). Interestingly, in the same study, minipigs vaccinated with capsular antigens, which are known to protect naive mice but failed in clinical trials (24), also failed to protect the minipigs from wound infection even though the vaccine elicited anti-capsule antibodies (23). It would be interesting to evaluate the α2,3 sialylation status of these anti-capsular antibodies. The use of minipigs as an alternate model for vaccine development and preclinical studies also highlights the power of natural infection models to gain insight into human pathophysiology. Second, would colonization elicit similar B cell imprinting as prior infection? Follow-up studies are needed to compare prior infection versus colonization and their impact on IL-10 production and immune imprinting. Lastly, an exciting avenue of future research is the idea of developing adjuvants and/or other strategies aimed at subverting the IL-10–imprinting response. If achievable, this will likely improve the vaccine response and get us closer to the Holy Grail — a *S*. *aureus* vaccine that works in humans.

Altogether, Tsai et al. (20) not only provides us with much needed mechanistic insight into why the IsdB vaccine, and potentially other OPK-promoting vaccines, have failed in human clinical trials, but also emphasizes the need to use preclinical models that better reflect the human situation. Overlooked in the field is that, in addition to humans, *S*. *aureus* is a major pathogen of animals, including cows, pigs, and rodents (25). These animal-adapted strains more faithfully recapitulate the tug of war between *S*. *aureus* and the mammalian host and overcome the pesky species-specificity limitation of many *S*. *aureus* virulence factors. While the numerous failed clinical trials might discourage research and development of the elusive anti–*S*. *aureus* vaccine, studies like that of Tsai et al. (20) in this issue of *JCI* provide a glimmer of hope. The study’s findings showcase that better-designed preclinical models and innovative antigen compositions could ultimately triumph against one of infectious diseases’ Goliaths.

## Version 1 12/16/2024

Electronic publication
